# Early Intervention as a Way of Reducing Neurocognitive Delay in Pediatric Sickle Cell Patients

**DOI:** 10.7759/cureus.97228

**Published:** 2025-11-19

**Authors:** London Wilson, Suzanne I Riskin

**Affiliations:** 1 Department of Foundational Sciences, Nova Southeastern University Dr. Kiran C. Patel College of Osteopathic Medicine, Clearwater, USA

**Keywords:** developmental delay, early intervention, early intervention services, neurocognitive delay, occupational therapy, pediatric, physical therapy, sickle cell anemia in children, sickle cell disease, speech therapy

## Abstract

Sickle cell disease (SCD) is a genetic blood disorder that largely affects African Americans in the United States. This disease leads to an increased risk of neurocognitive decline and delay in pediatric patients with or without cerebral events. Despite its proven benefits, early intervention (EI) is underutilized in this population. This review aims to investigate the use of specific EI services within the pediatric SCD population and whether any combination of these services can lead to a better outcome than others. A scoping review was conducted using PubMed, Excerpta Medica Database (Embase), and Cumulative Index to Nursing and Allied Health Literature (CINAHL) following the Preferred Reporting Items for Systematic Reviews and Meta-Analyses (PRISMA) guidelines. The methods involved a systematic search using Boolean-modifier terms related to sickle cell and neurocognitive delay, with inclusion and exclusion criteria applied to select 19 relevant pediatric-focused articles, guided by PRISMA methodology. Results found that the use of EI services has proven to be beneficial in this population. Further research is needed to investigate EI services, such as the use of these therapies for preventative measures prior to symptoms or diagnosis of neurocognitive delay, and whether the starting age could affect the neurocognitive outcomes of the patients.

## Introduction and background

Sickle cell disease (SCD) is an inherited blood disorder affecting more than 70,000 Americans, affecting 8% of the African American population, but also affecting Hispanic Americans from Central and South America and people of Middle Eastern, Indian, and Mediterranean descent [1]. SCD results from a point mutation replacing glutamic acid with valine in the beta-chain of hemoglobin, which alters the structure of hemoglobin. This change from a hydrophilic to a hydrophobic amino acid leads to hemoglobin aggregation, which can damage the red blood cell membrane and result in both extravascular and intravascular hemolysis. SCD occurs when two abnormal beta genes are present: upon deoxygenation, the hemoglobin aggregates into thin structures, causing the red blood cells to become sickle-shaped. Vaso-occlusive crises occur when sickling red blood cells become stuck on one another and within vessels, which blocks blood flow to areas downstream of the sickle [1]. 

Sickled cells aggregate due to deoxygenation and affect children and adults by causing complications such as acute chest syndrome and cerebral infarction. The neurological complication of SCD happens in up to 50% of SCD patients, with ischemic strokes occurring in up to 11% of pediatric patients and silent cerebral infarcts happening in 39% of pediatric patients by the time they are 18 years of age [2]. With these vascular events, there is the possibility of neurocognitive decline in children due to lack of blood flow to the brain, but it is not known if any one of these complications leads to an increased risk of delay more than another. It has recently been shown that even with no vascular events, the risk for neurocognitive decline is still present in children with SCD [3,4]. SCD patients present with lower full-scale IQ, academic achievement, and scores on cognitive function tests [4]. Neurocognitive delay screening is not specific to SCD patients and therefore has no standardized protocol; there is inconsistent neurocognitive screening in pediatric SCD patients [5]. Physicians often use the Ages and Stages Questionnaire (ASQ) or the Modified Checklist for Autism in Toddlers (MCHAT) to screen for moderate developmental delay, but can also use other tools such as the Communication and Symbolic Behavior scale (CSBS) and Denver Developmental Screening Test (DDSS) [5]. These tools screen for development without a cognitive focus and look at communication, gross motor, fine motor, problem-solving, and personal-social aspects [5]. This paper presents a scoping review to explore the use of early intervention (EI) services in SCD patients and evaluates the combination of these services to assess if neurocognitive outcomes are affected. EI services, including physical therapy, occupational therapy, and speech therapy, are proven to limit the neurocognitive deficits of young children with SCD and improve quality of life [6,7]. There has not been further research done to discover if a certain combination of these therapies can lead to better outcomes or if starting EI services before the start of the neurocognitive decline could lead to enhanced developmental progress.

## Review

Methods

Search Strategy

**Figure 1:**
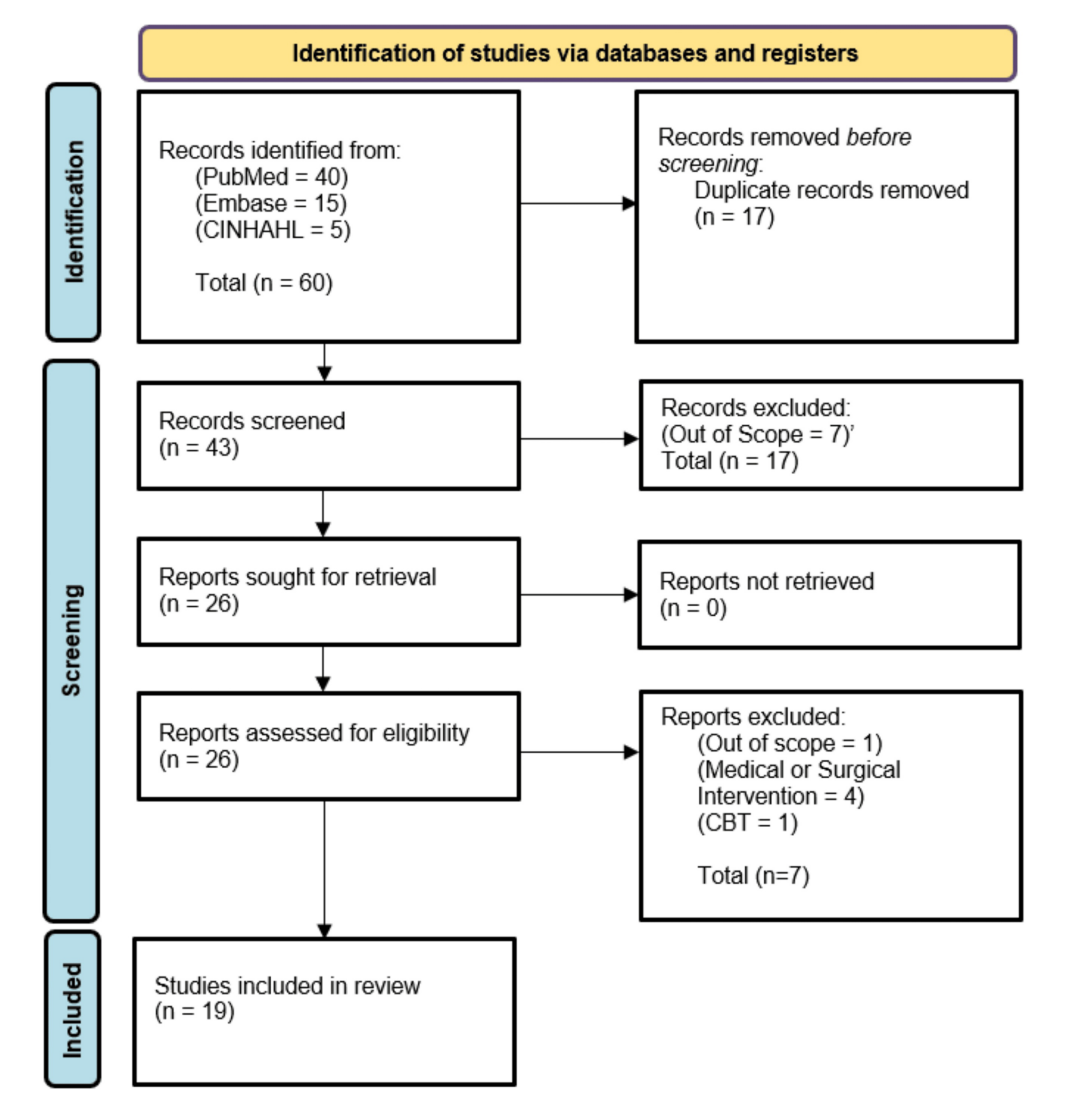
Preferred Reporting Items for Systematic Reviews and Meta-Analyses (PRISMA) diagram of the search strategy

The search was performed using three databases: PubMed, Excerpta Medica Database (Embase), and Cumulative Index to Nursing and Allied Health Literature (CINAHL). Search terms used with Boolean modifiers were: "sickle cell", "early intervention", "neurocognitive", "developmental delay", "early cognitive intervention services", "sickle cell", "sickle cell disease", "sickle cell anemia", "neurocognitive delay", "developmental decline", and "neurodevelopmental delay". These searches yielded 60 results, and 19 articles were selected for this paper. Inclusion criteria focused on pediatric sickle cell patients, the use of early intervention services, and the risk of neurocognitive decline or delay. Exclusion criteria were papers that used the term "early intervention" but were discussing medications or surgical interventions used, not EI services, papers discussing thalassemia and other sickle cell trait-related disorders, but not about SCD, and papers that were discussing cognitive behavioral therapy since it is not included in EI services. Figure 1 demonstrates the use of the Preferred Reporting Items for Systematic Reviews and Meta-Analysis (PRISMA). Sixty articles were identified, 17 were removed as duplicates, 17 records were excluded based on screening, seven more papers were excluded based on eligibility assessment, and 19 papers were used within this review.

Results

Neurocognitive Risk in Sickle Cell Disease

One of the main reasons SCD leads to neurocognitive delay is occlusion from sickled blood cells. Young children during a developmental age carry an increased risk of cerebral vascular infarcts, in which the events themselves lead to neurocognitive issues within the patients [3,4,8]. Almost 50% of sickle cell patients will have some form of cerebrovascular disease by 14 years of age [2]. Children with SCD are more susceptible to cognitive deficits, whether they have a previous cerebrovascular event or not, when compared to children without SCD [3]. There is a recent debate over whether these cognitive deficits are just a delay or rather a full decline overall, being called "a delay with decline" [9]. Delays in processing speeds of SCD patients worsen with age. The decline was largest for children less than 8.88 years of age [9]. Processing speeds decline in SCD patients and may be a target for treatment. SCD patients have a delayed onset in Verbal Comprehension Index (VCI), and executive function develops at a slower rate, making both VCI and executive function targets to treat cognitive delays [10]. Parental cognition and stress can affect the progress and decline of the patient's neurocognitive abilities. Increasing knowledge for parents about child development via physician education could decrease distress. Changing "learned helplessness" attributional styles could increase the cognitive outcomes of children with SCD [11].

Barriers to Screening and Access to Early Intervention Services

A study has shown that screening for language and cognitive development in SCD patients can help predict the outcome of their future academics and stroke risk, but this is not helpful if the children are not properly screened [12]. Developmental screening has proven to be beneficial in identifying these risks, but the screening is often inconsistent with no standardization, as well as not specific to SCD [5]. It has been found that between specialists such as hematologists and primary care physicians, there is inconsistency in who they believe should be checking for these neurocognitive issues. Pediatricians believe that SCD patients spend more time with their hematologist, who should know the signs and symptoms of the disease, while hematologists report that screening for neurocognitive delay should be done first by a primary care provider [6].

EI services, while proven to be effective in children with neurocognitive delays, are not used as often as they should in pediatric SCD patients. This is often due to the lack of awareness from caregivers about the risk of developmental delays, a lack of access to these services, and a lack of communication by providers about these services [6]. There is also the issue of hematologists and primary care providers both believing the other should be checking the patient for delays and referring them to these services [6]. The screening process for developmental delays can be overwhelming, so caregivers cannot bring their child to both their primary care and hematology appointments [13]. One other large factor in EI is limited access to the services. Providers do not always recommend EI services, and caregivers have limited knowledge about these services. Financial barriers arise, again noting that knowledge of these services and the neurocognitive risk is lacking [14]. The American Society of Hematologists (ASH) combats this risk by suggesting that a psychiatrist or pediatrician should screen pediatric SCD patients to check for this increased risk of cognitive decline. The inconsistency regarding who should be referring these patients often poses an issue [5,15]. Studies performed about the use of EI services in SCD patients as a home-based model found that recruitment and retention of families was an issue, as well as caregivers of patients older than eight months of age were less likely to partake in these services than caregivers of younger infants [16]. One occupational therapy study found that referring SCD patients to occupational therapy early can improve academic outcomes by looking at fine motor performance and math fluency [17]. The age at which SCD patients are screened and referred to EI services is important, as early identification and referral should be done as soon as possible [18].

Early Risk Factors to Address the Need for Early Intervention Services

Studies have shown how to identify risks for neurocognitive decline early, aside from screening by a pediatrician or hematologist. The catechol-O-methyltransferase (COMT) gene is a method to identify the risk of neurocognitive decline [19]. The COMT gene regulates the amount of dopamine available in the prefrontal cortex. The dopamine regulation by the COMT gene influences pain perception and frequency of vaso-occlusive crises in SCD patients, as well as is in neurocognitive function in the pediatric SCD population [19]. The polymorphisms found in the COMT gene are found to modulate dopamine, with a decreased level of dopamine leading to an increased neurocognitive deficit [19]. The COMT gene could be used to identify SCD patients at higher risk of neurocognitive decline and could imply the earlier need and use of early intervention services [19]. This study has a limitation in that the association was only found with male SCD patients and not female SCD patients [19]. Studies using fetal hemoglobin, a modifier of the phenotype of SCD, predict the neurocognition of SCD patients [20]. Low fetal hemoglobin has been associated with low IQ in SCD patients and can affect neurocognitive outcomes due to increased risk of polymerization, which increases the risk of sickling and vaso-occulsive events [20]. Studies on birth weight, gestational age, and neonatal intensive care unit admission identify ways that children diagnosed with SCD are more likely to need early intervention services [21]. Low birthweight of less than 2500 grams has lower working memory, while increased birthweight has increased processing speeds. SCD patients admitted to the neonatal intensive care unit had lower processing speeds and working memory. Increased gestational age is associated with higher working memory scores [21]. This study identifies low birth weight, gestational age, and history of neonatal intensive care unit as a new way to stratify SCD patients at higher risk of neurocognitive delay, and identify patients at an earlier age who could benefit from EI services [21].

Discussion

Both the risk of neurocognitive delay and the use of EI services are researched topics within the SCD population. It has been found that there is an increased risk in SCD patients with or without vaso-occlusive and cerebrovascular events [3]. Parental style, stress, and cognition all contribute to the risk of neurocognitive delay in SCD patients and should be looked at as factors when identifying high-risk SCD patients [11]. Screening in SCD patients poses as a barrier to patients getting EI services, as screening is often inconsistent, not disease specific, and not as thorough as pediatricians and hematologists have discourse over who should be screening SCD patients. While EI services, including physical therapy, occupational therapy, and speech therapy, are proven to be beneficial in pediatric SCD patients, there is a lack of utilization of these services [6]. There is also research proving that starting EI services at an earlier age leads to improved neurocognitive outcomes, but there is no research on if starting EI services for patients at high risk for neurocognitive delay, before the delay is prevalent, could lead to an increase in outcomes [6,7]. Using risk factors such as genetics, the catechol-O-methyltransferase gene, and the fetal hemoglobin gene, as well as early factors such as birth weight, gestational age, and neonatal intensive care unit admission, pediatric SCD patients can be identified who are at a higher risk and require EI services [21]. There could be future research done on starting EI services before any delay or decline is apparent in screened patients who have identified risks. There is a lack of research on the uses of the different types of EI services and the changes in neurocognitive decline. While EI services are typically tailored to each child, knowing the proven risk of possible developmental delays means patients could benefit from multiple therapies, physical, occupational, and speech, rather than just one.

## Conclusions

SCD is a complex, multigenic blood disorder that can lead to the development of neurocognitive deficits in pediatric patients with or without cerebral events caused by the disease. EI services are well-studied within this population and have been proven to reduce the risk of the severity of neurocognitive decline or delay. However, further research is still to be done to discover whether a certain combination of these services is more effective, and if using newly identified factors to stratify patients as high risk and begin patients on EI services before a neurocognitive delay becomes apparent could affect the neurocognitive outcomes of patients. There is a need for future research to be done to identify new standards to increase the quality of life of pediatric sickle cell patients.
